# Current Challenges of Managing Fibrosis Post Glaucoma Surgery and Future Perspectives

**DOI:** 10.3390/jcm14238548

**Published:** 2025-12-02

**Authors:** Phey Feng Lo, Seok Ting Lim, Xiaomeng Wang, Tina T. Wong

**Affiliations:** 1 Singapore National Eye Centre, Singapore 168751, Singapore; 2 Singapore Eye Research Institute, Singapore 169856, Singapore; 3 Centre for Vision Research, Duke-NUS Medical School, Singapore 169857, Singapore

**Keywords:** fibrosis, glaucoma filtration surgery, wound healing

## Abstract

The primary cause of post-operative failure following glaucoma filtration surgery is excessive bleb scarring. Traditional anti-fibrotics such as Mitomycin C (MMC) and 5-fluorouracil (5-FU) have greatly improved bleb survival but are not without their complications. Insights gained from traditional trabeculectomy studies can be directly applied to modern minimally invasive glaucoma surgery (MIGS) techniques. As surgical techniques continue to advance and overall safety improves, there is a growing need to explore other novel therapeutics that offer increased efficacy and favourable safety profiles. This review aims to provide insight into the pathophysiology of wound healing as well as discuss current and emerging strategies being developed to address wound healing post glaucoma filtration surgery.

## 1. Introduction

Glaucoma remains the leading cause of irreversible blindness worldwide, with global prevalence projected to rise to 111.8 million by 2040 [1]. This growing burden contributes significantly to economic costs and societal impact due to the high rates of visual impairment associated with the disease [1]. Despite advancements in medical therapies and surgical techniques, glaucoma affected an estimated 4.14 million people in 2020 alone, resulting in moderate-to-severe visual impairment [2].

To date, intraocular pressure (IOP) remains the only modifiable risk factor for glaucoma. IOP is determined by the equilibrium between aqueous humour production and drainage [3]. Aqueous humour exits the eye via two primary pathways: the conventional outflow pathway, which passes through the trabecular meshwork into Schlemm’s canal, collector channels and episcleral veins, and the unconventional (uveoscleral) pathway, which traverses the ciliary body band through to the suprachoroidal space and out through the sclera [3]. Changes in extracellular matrix (ECM) composition, increased trabecular meshwork contractility and disruption of local regulatory mediators lead to resistance in aqueous humour outflow [3].

Surgical interventions for glaucoma aim to reduce IOP by enhancing aqueous outflow. Traditional bleb-forming procedures, such as trabeculectomy and glaucoma drainage devices, create an alternative drainage route into the subconjunctival space [4]. Over the past decade, surgical approaches in glaucoma have evolved significantly. Traditional glaucoma filtration procedures are increasingly being replaced by safer and more effective techniques [5]. More recently, minimally invasive bleb-forming approaches such as Minimally Invasive Bleb Surgery (MIBS), have emerged as additional options to facilitate subconjunctival filtration, bypassing the native aqueous humour outflow pathway [4].

However, the long term success of any glaucoma filtration surgery remains limited by post-operative scarring, with fibrosis at the filtering bleb being a primary cause of surgical failure [6]. Despite innovative surgical advances, the presence of anti-fibrotic factors affects long-term outcomes. Newer MIBS procedures present challenges in post-operative management and often require a higher concentration of traditional anti-fibrotic agents, such as Mitomycin C (MMC), applied to the tissues for a longer period of up to 5 min to inhibit the fibrotic response. The wound healing process at the surgical site, local tissue remodelling, systemic immune responses to injury and the pro-inflammatory composition of aqueous humour filtering into the bleb remain areas that warrant further investigation. To achieve long-term surgical success alongside development of new anti-scarring therapeutic strategies, a comprehensive understanding of the role these mechanisms play in modulating post-operative wound healing response is essential. This review aims to summarise the mechanisms of wound healing as well as discuss both current and emerging strategies currently being developed to prevent fibrosis in bleb-forming surgeries.

## 2. Wound Healing and the Conjunctival Fibrotic Response

**Figure 1:**
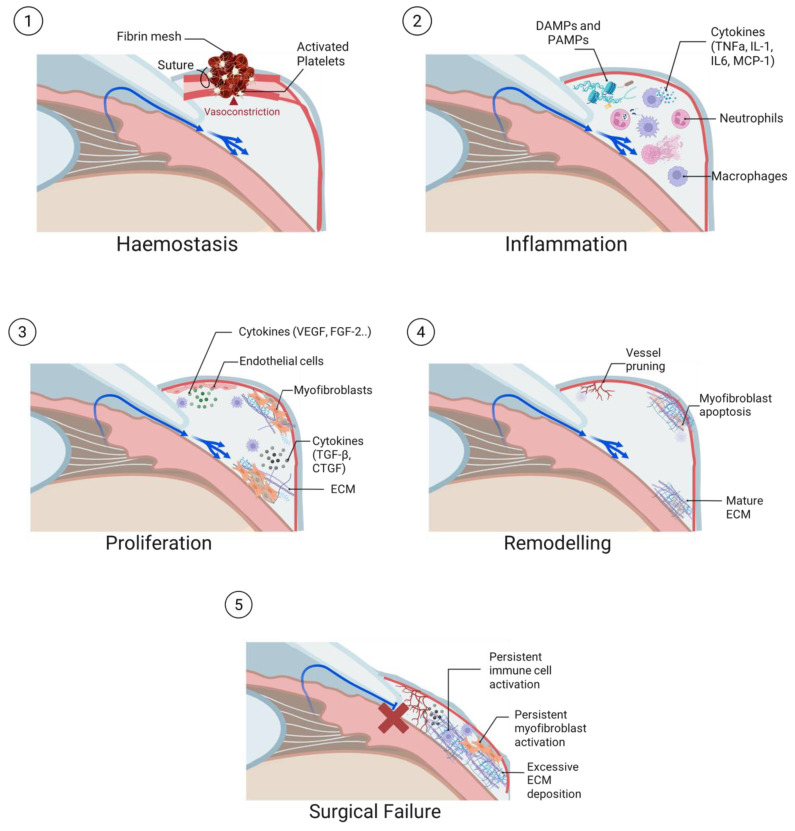
Stages of wound healing post-trabeculectomy. (**1**) Haemostasis, where the damaged vasculature undergoes vasoconstriction and platelets are activated to culminate in the formation of a fibrin mesh (Note that a portion of the vessel has been enlarged for illustrative purposes). (**2**) Inflammation, where the presence of danger signals such as DAMPs and PAMPs results in the infiltration of immune cells like neutrophils and macrophages to the bleb site. (**3**) Proliferation, where fibroblasts are activated to become myfibroblasts and ECM is deposited. Endothelial cells also undergo proliferation and migration to form new vessels. (**4**) Remodelling, a process where activated myofibroblasts, immune cells and endothelial cells undergo apoptosis, and the ECM matures. (**5**) Surgical failure occurs in the event of excessive post-operative fibrosis. In certain individuals, uncontrolled activation of immune cells and myofibroblasts results in accumulation of ECM, thus impeding the outflow of aqueous humour and resulting in bleb failure.

The post-operative conjunctival wound healing process can be divided into four key stages: haemostasis, inflammation, proliferation and tissue remodelling (Figure 1) [7]. Each stage involves a dynamic interplay between different cell types, growth factors and cytokines, and when dysregulated, can lead to excessive subconjunctival fibrosis and surgical failure [8].

### 2.1. Haemostasis

Haemostasis is a multi-step process that occurs immediately following surgery-induced wounding. During filtration surgery, the incisional procedures performed on the conjunctiva and sclera result in connective tissue and vascular damage [9]. Vasoconstrictors such as endothelin-1 are released by the damaged vasculature to promote rapid and reflexive contracture of the vascular smooth muscle cells to minimise bleeding [10,11]. This process leads to platelet adhesion, activation and plug formation [10]. Activated platelets produce substances such as thromboxane A2 that can further promote vasoconstriction and amplify the initial response [12]. This process leads to the subsequent initiation of a blood coagulation cascade [13,14,15] and ultimately culminates in the formation of a fibrin clot to prevent excessive blood loss [7,16]. Platelets concurrently release growth factors like transforming growth factor-alpha (TGFα), transforming growth factor-beta (TGFβ), platelet-derived growth factor (PDGF) and Platelet Factor 4 (PF4) [7,14] which can contribute to inflammatory cell infiltration to the site of injury.

### 2.2. Inflammation

The inflammatory phase following haemostasis lasts between one to six days post-wounding. This phase is initiated by the presence of two types of danger signals detected in the surgical wound: damage-associated molecular patterns (DAMPs) and pathogen-associated molecular patterns (PAMPs) [17]. DAMP molecules are endogenous cellular molecules such as nucleic acids, proteins and metabolites released by injured host tissues while PAMPs are non-host molecules belonging to invading pathogens such as bacterial lipopolysaccharides and nucleic acids [18]. Both DAMPs and PAMPs are recognised by pattern-recognition receptors (PRRs) such as Toll-like receptors to trigger an influx of immune cells such as neutrophils and macrophages to the bleb site [7,17].

Neutrophils are typically the first to be recruited to the site of damage, where they function to remove necrotic tissue debris and pathogens via phagocytosis and NETosis (release of neutrophil extracellular traps, NETs). Thereafter, circulating monocytes infiltrate the wound, where they differentiate into tissue macrophages to further augment the inflammatory response [19]. Macrophages secrete various pro-inflammatory soluble factors such as tumour-necrosis factor-alpha (TNFa), interleukins (IL) like IL-1, IL6 and IL-8, monocyte-chemoattractant protein-1 (MCP-1) and macrophage colony-stimulating factor (M-CSF) [14,20]. These factors are crucial for the recruitment of other inflammatory cells and the activation of fibroblasts [7,16], which are the main cellular effectors for the next phase of wound healing.

### 2.3. Proliferation

The proliferation phase of wound healing is characterised by the formation of highly vascularised granulation tissue in the wound bed. In this phase, quiescent fibroblasts residing primarily in the conjunctiva and Tenon’s capsule proliferate and undergo transdifferentiation to become activated myofibroblasts. Myofibroblasts deposit extracellular matrix (ECM) and express contractile proteins like α-smooth muscle actin (α-SMA) to facilitate wound contraction [7,13,15]. Proteolytic enzymes such as matrix metalloproteinases (MMPs) are also produced to enable fibroblast migration to the wound bed [16]. In ocular tissues, all three isoforms of TGFβ, especially TGFβ2, have been shown to be central to this process [13,20]. Predominantly produced by infiltrating macrophages and fibroblasts, TGFβ binds to transmembrane serine/threonine kinase TGFβ receptors to activate both canonical Smads [21] and non-canonical signalling pathways such as mitogen-activated protein kinase (MAPK) and c-Jun-N-terminal kinase (JNK) [20]. Activation of these pathways stimulates fibroblast proliferation, migration and mRNA upregulation of both structural proteins like collagen and fibronectin, and matricellular/non-structural proteins such as secreted protein acidic and rich in cysteine (SPARC) [15]. Importantly, TGFβ can also stimulate the expression of other pro-fibrotic mediators such as Connective Tissue Growth Factor (CTGF) to further amplify this process [13,15].

Concurrently, microvascular endothelial cells (ECs) are also activated in this phase of wound healing, where they initiate sprouting angiogenesis in response to secreted pro-angiogenic signals such as vascular endothelial growth factor (VEGF), fibroblast growth factor-2 (FGF-2), PDGF-β and angiopoietins, leading to EC proliferation and migration [7,14].

### 2.4. Tissue Remodelling

In the final stage of the wound healing response, the original granulation tissue is gradually supplanted by newly formed connective tissue [7]. This remodelling phase is typically characterised by blood vessel regression [13], massive apoptosis of activated fibroblasts [7,16] and immune cells [14], and the replacement of Type III collagen by cross-linked Type I collagen. This eventually results in increased tensile strength and elasticity of the healed tissue [7] and mature scar formation.

### 2.5. Conjunctival Fibrotic Response in Trabeculectomy

The entire wound healing process following a trabeculectomy, starting with haemostasis to tissue remodelling, can last over a period of several months to up to 2 years [7]. Unlike other surgical procedures where a complete healing and functional restoration of the tissue is desired, the success of a trabeculectomy in achieving intraocular pressure (IOP) reduction relies on the precise modulation of wound healing to enable the optimal flow of aqueous humour from under the scleral flap into the subconjunctival space [7,9]. In certain individuals, persistent subconjunctival and episcleral fibrosis reminiscent of a non-healing wound can progressively compromise aqueous humour drainage through the sclerostomy, leading to bleb failure [21].

The hallmark of pathological fibrosis is the excessive accumulation of ECM. While mechanisms contributing to conjunctival scarring are not completely understood [21], known risk factors include previous history of conjunctival surgical procedures, long-term topical conjunctival medication, ocular surface inflammation, ethnicity and young age [15]. In essence, the normally efficient wound healing process becomes overactive and dysregulated; and the conjunctival epithelium experiences a state of constant, chronic inflammation characterised by uncontrolled production of both pro-inflammatory and pro-fibrotic growth factors and cytokines [7]. This leads to the persistent activation of myofibroblasts and the continual deposition of ECM [13], and when left unchecked, would disrupt aqueous humour drainage into the bleb and impede its final absorption in the subconjunctival space, leading to bleb failure [20]. Indeed, trabeculectomy failures have often been associated with marked inflammatory responses and increased fibroblast counts [14]. Consequently, strategies that suppress inflammation and limit fibroblast activation are critical in ensuring the success of GFS.

## 3. Strategies to Prevent Fibrosis Following Glaucoma Filtration Surgery

Anti-inflammatory drugs such as non-steroidal anti-inflammatory drugs (NSAIDs) and corticosteroids target the inflammatory phase of the wound healing response. Steroids suppress the cellular inflammatory response by reducing concentration, migration and activity of neutrophils and macrophages. They inhibit phagocytosis, growth factor release and antigen responses. Intracellularly, steroids block the conversion of membrane phospholipids into arachidonic acid, preventing the initial step of downstream inflammatory mediators such as leukotrienes, prostaglandins and thromboxanes. In addition, glucocorticoids lower vascular permeability, limiting the release of growth factors, clot formation and cell migration to the injury site [22].

Topical steroids are the most commonly used form of corticosteroid therapy following glaucoma filtration surgery, followed by subconjunctival and oral preparations. These are typically administered intra-operatively and post-operatively. In addition, pre-operative steroid use has also been shown to enhance surgical outcomes. Broadway et al. demonstrated that starting topical steroid drops one month prior to surgery, after discontinuing anti-glaucoma medications, led to a reduction in conjunctival fibroblasts and inflammatory cells [23]. This approach resulted in improved surgical success rates from 50% to 81% [23]. A more recent study in Asian eyes demonstrated patients with a high level of the pro-inflammatory cytokine monocyte chemoattractant protein-1 (MCP-1) benefitted from a 2-week pre-operative course of steroids [24]. This improved early post-operative outcomes in patients undergoing trabeculectomy and phaco-trabeculectomy [24]. Whilst steroids have proven efficacy, there has been no established consensus on the optimal dosing or duration of treatment. Their main challenges include systemic side effects, increased risk of cataract formation and more notably, raised intraocular pressure. Steroid-induced IOP elevation typically occurs after several weeks of continuous therapy and generally resolves upon discontinuation of the medication. Risk factors for developing steroid-induced glaucoma include diagnosis or first degree family history of primary open-angle glaucoma, type 1 diabetes mellitus, high myopia and certain connective tissue diseases like rheumatoid arthritis [25].

NSAIDs act further downstream in the inflammatory pathway by inhibiting cyclooxygenase. NSAIDs are associated with a more favourable side-effect profile compared to steroids, with emerging evidence reporting their potential role in modulating post filtration surgery outcomes. A study by Sim et al. found that adjunctive use of oral ibuprofen in patients with a high risk of early bleb failure post trabeculectomy resulted in greater IOP reduction and a lower incidence of bleb failure compared to patients not receiving ibuprofen [26]. These findings align with other studies suggest that combining NSAIDs with steroids produce a synergistic anti-inflammatory effect. This was demonstrated by Fuller et al., where oral steroids and NSAIDs were given to patients exhibiting signs of bleb failure post trabeculectomy [27]. Their study reported a significant reduction in bleb vascularity, improved bleb diffusion and subsequent IOP reductions [27].

Is one better than the other? In a randomised clinical trial by Breusegem et al., participants were assigned to one of three groups—placebo, topical NSAIDs and topical steroids [28]. Each group instilled one drop of their medication four times a day for one month prior to undergoing glaucoma filtration surgery. Both the NSAID- and steroid-treated group required significantly fewer post-operative bleb needlings compared with placebo [28]. None of the patients in the steroid group required further IOP-lowering drops to maintain the target IOP at 1 year [28]. These findings suggest that that the pre-operative modulation of inflammation with either NSAIDs or corticosteroids may enhance the success of filtration procedures. Conversely, a randomised controlled trial comparing ketorolac with dexamethasone in patients undergoing Ahmed valve surgery demonstrated that mean IOP was consistently higher in the steroid group, with a statistically significant difference observed at week 4 [29]. This raises the possibility that corticosteroids may contribute to the hypertensive phase commonly seen after glaucoma drainage device surgery. The NSAID group did however have a higher incidence of conjunctival retraction compared with the steroid arm [29]. These findings suggest differing effects on wound modulation post-surgery and warrant further investigation.

Acting further upstream of the wound healing cascade, anti-fibrotics prevent fibroblast replication and production of collagen and scarring. Some of the most utilised anti-fibrotic agents in glaucoma filtration surgery include Mitomycin C (MMC) and 5-fluorouracil (5-FU). Mitomycin C is an alkylating agent that inhibits DNA synthesis during the G1 and S phases of the cell cycle, reducing cell proliferation [30]. Apoptosis has been observed in cultured human Tenon’s fibroblasts following MMC administration, with a dose-dependent increase in apoptotic activity occurring approximately 48 h post exposure [31]. MMC was first introduced into glaucoma in the early 1990s as an adjunct to trabeculectomy. Its use resulted in long-term IOP control by delaying post-operative wound healing through inhibition of fibroblast proliferation [30]. More recent surgical techniques like MIBS including the Preserflo microshunt and the Xen gel stent, also rely on the use of MMC to improve surgical outcomes. Despite advances in modern surgical techniques, we still heavily rely on the use of traditional adjunctive anti-fibrotics to achieve surgical success. However, the optimal concentration and exposure time of MMC remains debatable. In addition, evidence supporting the benefit of MMC in conjunction with aqueous shunt surgery is limited [32]. A recent study reported that MMC use was associated with lower IOP during the first 6 months, however this difference was not significant by month 12, although the overall IOP remained lower in the MMC group [33]. MMC also blunted the hypertensive phase but was associated with a slightly higher incidence of transient hypotony [33]. These findings suggest that MMC should be used judiciously in glaucoma drainage device surgery.

5-FU is a chemotherapeutic agent and acts by antagonising pyrimidine metabolism leading to cell death. Experimental models have shown that short exposures to 5-FU inhibited ocular fibroblast-mediated collagen contraction [34]. This led to translation to clinical use where 5-FU was used intra-operatively or in the post-operative setting of imminent bleb failure [22]. A multi-centre clinical trial by the 5- Fluorouracil Filtering Study Group was a randomised prospective study on the use of 5-FU in high-risk trabeculectomy patients [35]. Patients were randomised to receiving trabeculectomy alone or trabeculectomy with twice-daily subconjunctival 5-FU injections for a week followed by daily injections for a week. The failure rate at 5 years was significantly lower in the treated group at 51% vs. 74%. However, there was a higher incidence of bleb-related complications than the control group. The group recommended the use of 5-FU in high-risk eyes but caution in eyes that were of lower risk [35].

To date, the use of anti-fibrotics is common intra-operatively and in the post-operative setting of ‘at risk’ blebs. However, its use is not without complications. These include bleb leaks, avascular or cystic blebs, choroidal effusions and hypotony. As anti-metabolites are in liquid form, injection carries the risk of leakage from the injection site, leading to extraocular injury, or more seriously, intraocular injury. On the other hand, sponge delivery to the subconjunctival space makes it difficult to determine the dosage administered. Nevertheless, it has been proven that the intra-operative and post-operative use of MMC and 5-FU significantly reduces the risk of surgical failure [36].

## 4. Future Directions

### 4.1. Investigative Drugs

Current strategies for wound healing are effective; however, there remains a need for alternative approaches associated with fewer complications. One promising alternative is the use of monoclonal antibodies that target specific molecular markers, such as transforming growth factor-beta (TGFβ). Unlike MMC and 5-FU, which exert nonspecific mechanisms of action, monoclonal antibodies offer greater specificity. TGFβ2 is the most predominant isomer found in the eye and plays a central role in the fibrotic response associated with ocular scarring [37]. Cordeiro et al. investigated the efficacy and safety of recombinant human anti-TGFβ2 monoclonal antibody (rhAnti-TGFβ2 mAb) on ocular fibrosis [38]. In vitro studies demonstrated a significant reduction in fibroblast proliferation and migration while in vivo studies in a glaucomatous rabbit model showed improved outcomes following filtration surgery [38]. A further development, CAT-152, exhibited high affinity and specificity for TGFβ2. In a rabbit model, a post-operative regimen of seven subconjunctival injections of CAT-152 significantly improved surgical outcomes and bleb survival [39]. Despite these promising preclinical findings, a phase III clinical trial failed to show a significant difference between CAT-152 and placebo groups in preventing surgical failure [40]. The investigators postulated that dosing regimens from animal models may not have been fully optimised for human trials and that further studies adjusting for dosing strategies and delivery methods are warranted.

During the proliferative phase of wound healing, fibroblasts and angiogenesis are promoted by various growth factors such as vascular endothelial growth factor (VEGF). The upregulation of these growth factors have been associated with increased scar formation and a higher risk of bleb failure [41]. In vitro studies have demonstrated that bevacizumab, a monoclonal antibody targeting VEGF-A, effectively inhibits fibroblast proliferation, and reduces angiogenesis and collagen deposition [42]. A randomised clinical trial evaluating the intra-operative administration of a single intravitreal dose of bevacizumab in conjunction with standard MMC, compared to MMC with placebo, had results showing improved surgical success and a reduced need for medication or further surgical interventions at 12 months [43]. Similarly, intra-operative subconjunctival bevacizumab given alongside standard MMC achieved similar results at 6 months with an average IOP reduction of 52% [44]. Despite anti-VEGF agents showing promise in modulating wound healing, a longer-term randomised clinical trial is necessary to confirm their efficacy and safety. Additionally, exploration of newer VEGF inhibitors, some of which have demonstrated longer inhibitory activity in the treatment of retinal diseases, may offer further therapeutic potential in the context of glaucoma filtration surgery.

Beta radiation offers a non-pharmacologic method for modulating wound healing. It has been shown to inhibit fibroblast proliferation of human Tenon’s fibroblasts by arresting growth but not cell death through a mechanism involving increased p53 [45]. Constable et al. demonstrated that, unlike anti-fibrotics, beta radiation had no effect on cell migration or contraction [46]. However, it altered extracellular matrix production, which is a distinct mechanism of the wound healing pathway [46]. The advantage of this is the relatively low cost, ease of use and its long service life [47]. Disadvantages include it being a radioactive device, including the possibility of increased cataract formation and keratopathy [47]. More data comparing its safety and efficacy to liquid anti-fibrotics is warranted.

### 4.2. Preclinical Drugs

Valproic acid (VPA) is a drug widely used in the treatment of neurological diseases with additional repurposed anti-fibrotic properties identified. It has a unique property of being an effective inhibitor of type 1 collagen in conjunctival fibroblasts by modulating Smad expression pathways [48]. The ability to disrupt mature collagen formation and organisation reduces subconjunctival bleb fibrosis and is favourable towards bleb survival. In a rabbit MIBS model, a study comparing the efficacy of low dose MMC with VPA versus low and high dose MMC alone demonstrated that combination therapy effectively reduced collagen production whilst improving bleb vasculature [49]. These changes favoured the development of bleb morphology conducive to filtration. A subsequent study by the same group reinforced these findings and reported an excellent safety profile for the use of VPA [50]. The study also explored the use of topical VPA as an alternative mode of administration, offering flexibility in drug delivery across different phases of wound healing. Both subconjunctival and topical VPA delivery maintained desirable bleb characteristics and achieved comparable IOP reduction [50].

Non-SMAD pathways downstream of TGFβ signalling include those targeting PPARγ ligands, p38 MAPK inhibitors, JNK inhibitors, mTOR inhibitors and Rho signalling inhibitors. Of these, the most promising are ROCK (Rho-associated protein kinases) inhibitors. When activated, Rho binds to and activates ROCK, which phosphorylates downstream intracellular substrates [51]. Binding of ROCK regulates myoglobin/actin contraction, altering cell morphology and stiffness [51]. Ripasudil, a selective ROCK inhibitor that is currently in use in clinical practice, was shown to attenuate fibroblast expression in human conjunctival fibroblasts [52]. This suggests that Ripasudil may play a role in potentially inhibiting excessive scarring post glaucoma filtration surgery.

Secreted protein acidic and rich in cysteine (SPARC), a small molecule glycoprotein, is widely expressed in various tissues of the eye. It plays an important role in regulating cellular proliferation and migration of fibroblasts inducing collagen production. In systemic conditions such as lung fibrosis, skin fibrosis and pancreatic fibrosis, SPARC was shown to activate fibroblasts, increasing extracellular matrix production [53]. In the eye, the SPARC gene is highly expressed at sites of wound healing and tissue remodelling, including at wounded conjunctiva. This suggests a strong association between high levels of SPARC and fibrosis. Seet et al. utilised siRNA to suppress SPARC expression in conjunctival fibroblasts [54]. Compared to MMC, SPARC knockdown resulted in a similar reduction in cell proliferation [54]. However, MMC treatment led to a significantly higher increase in necrotic cell death, a cytotoxic effect that was not observed with SPARC silencing [54]. These findings suggest that SPARC knockdown modulates key wound healing processes without inducing cellular toxicity. A mouse model of conjunctiva scarring further evidenced that SPARC siRNA was able to effectively downregulate SPARC expression and improve bleb survival [55].

Macrophages are a key immune cell that are centrally involved in wound healing. M2 macrophages are a major source of TGFβ1, a key cytokine involved in fibrosis [56]. Yes-associated protein (YAP) and transcriptional coactivator with PDZ-binding motif (TAZ) are downstream regulators linked to the pathophysiology of fibrosis [56]. YAP/TAZ signalling have significant crossover with TGFβ1/Smad. Wang et al. hypothesised that YAP/TAZ is involved with the M2 fibrotic process in subconjunctival fibrosis. The study, although inconclusive, was promising, indicating that M2 macrophage may be a potential target for anti-fibrotic treatment [56].

Non-coding RNAs such as microRNAs (miRNAs) and long noncoding RNAs (lncRNAs) have been found to be promising in regulating bleb survival post glaucoma filtration surgery [57]. miRNAs are not only found in cells and tissues but also in tears and aqueous humour, ref. [57] and they play critical roles in regulating most cellular processes. To date, both pro-fibrotic (e.g., miR-200c [58], miR-216b [59]) and anti-fibrotic (e.g., miR-26a [60], miR-29b [61], miR-139 [62]) miRNAs have been identified using studies conducted in human Tenon’s fibroblasts. Depending on the function of the miRNAs, either miRNA mimics or anti-miRNA molecules packaged into novel polymeric vectors, viral vectors and lipid nanoparticles can been considered, although this method is often limited by the possibility of off-target side effects and the presence of pathway redundancies [57]. Several pro-fibrotic lcnRNAs such as H19, NR003923 and LINC00028 have also been identified, although their therapeutic potential remains to be further evaluated [57].

Monocyte chemoattractant protein-1 (MCP-1)/CCL 2 is important in monocyte recruitment. MCP-1 has been found to be associated with glaucoma progression where high levels of MCP-1 detected in the aqueous humour were associated with visual field progression in eyes with normal tension glaucoma [63]. Furthermore, high pre-operative MCP-1 levels found in the tears from subjects undergoing trabeculectomy were associated with higher rates of trabeculectomy failure [64]. Chong et al. evaluated the effect of inhibiting MCP-1 receptor on an experimental model of glaucoma filtration surgery [65]. Results showed prolonged bleb survival with less cellular toxicity compared to MMC [65]. Consistently, the use of anti-MCP-1 L-RNA aptamer, mNOX-E36, also demonstrated comparable efficacy to MMC in attenuating post-operative inflammation and fibrosis following GFS [66]. More recently, genetic knockout MCP-1 models showed preserved structural and limited functional retinal ganglion cells making it an interesting chemokine for future hypertensive models [67].

Matrix metalloproteinases (MMPs) are a family of enzymes that play a key role in all phases of wound healing. Fibroblasts and macrophages release MMPs to remodel initial, immature scar tissue. This enzyme, found in trabeculectomy bleb walls and tube shunts, degrades extracellular matrix (ECM) material allowing fibroblasts through newly granulated tissue. Ilomastat, an MMP inhibitor, prolonged bleb survival with reduced IOP lowering in a rabbit filtration surgery model [68]. Another group reported that Ilomastat given subconjunctivally was less toxic to ocular structures when compared with MMC [69]. Development of an Ilomastat–cyclodextrin eyedrop has shown promise with good solubility and permeability in ocular tissues, with retention of its in vitro activity. Doxycycline, another MMP inhibitor, was also compared to MMC in a rabbit post filtration surgery model. The group receiving topical doxycycline showed no difference in terms of bleb appearance or inflammation, having a similar efficacy to MMC [70,71]. Table 1 below summarises the therapeutic agents discussed above.

## 5. Conclusions

Glaucoma filtration surgery has undergone remarkable advancements in recent decades. Despite that, modulation of wound healing remains the key determinant of long-term surgical success. Emerging pharmacologic and technological innovations show promise for achieving efficacious IOP lowering with improved safety profiles that are important in optimising long-term surgical outcomes.

## Figures and Tables

**Table 1:** Summary of investigative and preclinical therapeutic adjunctive agents to improve the success of glaucoma filtration surgery.

Therapeutic Agents	Molecular Target	Mechanism of Action	Advantages	Potential Risks	Stage of Development
TGFβ antibody [38,39,40]	Inhibits TGFβ	Reduces fibroblast proliferation and migration	Greater specificity	Mild corneal staining	Phase III clinical trial of CAT-152 failed to show a significant difference
VEGF antibody [42,43,44]	Inhibits VEGF	Inhibits fibroblast proliferation, reduces angiogenesis and collagen deposition	Greater specificity	Avascular blebs, bleb-related complications, hypotony	Longer term randomised clinical trial required
Beta radiation [45,46]	Increases p53	Inhibits fibroblast proliferation alter ECM production	Low cost, long service life	Cataract formation, keratopathy	More safety and efficacy data required
Valproic acid [49,50]	Inhibits type 1 collagen	Suppresses pro-fibrotic Smad2/3/4 signalling, promotes anti-fibrotic Smad6 pathway	Also acts on collagen, resulting in a more favourable bleb morphology	None noted to date	In vivo studies on mouse and rabbit models of GFS have shown promising results
ROCK inhibitor [51,52]	Rho-kinase	Alters myoglobin/actin contraction, inhibits fibroblast proliferation	Greater specificity	Conjunctival hyperaemia, blepharitis, keratopathy	In vivo studies have shown promising results
SPARC siRNA [54,55]	Suppress SPARC expression	Suppresses fibroblast proliferation, less collagenous ECM	Improves bleb survival, no cellular toxicity	None noted to date	In vivo studies on mouse and rabbit models of GFS have shown promising results
YAP/TAZ signalling inhibitor [56]	M2 macrophages	Activates Smad 2/3, mediates TGFβ1/2	Greater specificity	None noted to date	In vitro studies inconclusive
miRNA mimics or anti-miRNA [57]	miRNA	Different miRNAs have pro- or anti-fibrotic effects	Selectively target genes	Unintended effect on other genes/limited impact on target genes	Mostly studies conducted in vitro on human Tenon’s fibroblasts
MCP-1 inhibitors [65,66]	CCR2 receptor antagonist or MCP-1 aptamer	Inhibits MCP-1 activity	Improves bleb survival, less cellular toxicity	None noted to date	In vivo studies on mouse models of GFS show promising results
MMP inhibitor [68,69,70,71]	MMP	Degrades ECM	Improves bleb survival, reduces scar tissue	Mild conjunctival toxicity	In vivo studies on rabbit models of GFS show promising results

## Data Availability

No new data were created or analysed in this study. Data sharing is not applicable to this article.

## Abbreviations

The following abbreviations are used in this manuscript:

IOP	Intraocular pressure
ECM	Extracellular matrix
MIBS	Minimally Invasive Bleb Surgery
MMC	Mitomycin C
TGFα	Transforming growth factor-alpha
TGFβ	Transforming growth factor-beta
PDGF	Platelet-derived growth factor
PF4	Platelet Factor 4
DAMP	Damage-associated molecular patterns
PAMP	Pathogen-associated molecular patterns
PRR	Pattern-recognition receptors
NETs	Neutrophil Extracellular Traps
TNFα	Tumour-necrosis factor-alpha
IL	Interleukins
MCP-1	Monocyte-chemoattractant protein-1
M-CSF	Macrophage colony-stimulating factor
α-SMA	α-smooth muscle actin
MMP	Matrix metalloproteinases
MAPK	Mitogen-activated protein kinase
JNK	c-Jun-N-terminal kinase
SPARC	Secreted protein acidic and rich in cysteine
CTGF	Connective Tissue Growth Factor
EC	Endothelial cells
VEGF	Vascular endothelial growth factor
FGF-2	Fibroblast growth factor-2
NSAIDs	Non-steroidal anti-inflammatory drugs
5-FU	5-fluorouracil
VPA	Valproic acid
ROCK	Rho-associated protein kinases
YAP	Yes-associated protein
TAZ	Transcriptional coactivator with PDZ-binding motif
miRNA	microRNA
lncRNA	Long non-coding RNA
